# Langmuir-Blodgett Films of Supported Polyester Dendrimers

**DOI:** 10.5402/2012/906839

**Published:** 2012-10-16

**Authors:** Rocío Redón, M. Pilar Carreón-Castro, F. J. Mendoza-Martínez

**Affiliations:** ^1^Centro de Ciencias Aplicadas y Desarrollo Tecnológico, Universidad Nacional Autónoma de México, Cd. Universitaria A.P. 70-186, 04510 Coyoacán, DF, Mexico; ^2^Instituto de Ciencias Nucleares, Universidad Nacional Autónoma de México, Cd. Universitaria A. Postal 70-543, 04510 Coyoacán, DF, Mexico

## Abstract

Amphiphiles with a dendritic structure are attractive materials as they combine the features of dendrimers with the self-assembling properties and interfacial behavior of water-air affinities. We have synthesized three generations of polyester dendrimers and studied their interfacial properties on the Langmuir films. The behavior obtained was, as a rule, the lowest generation dendrimers behaving like traditional amphiphiles and the larger molecules presenting complicated isotherms. The Langmuir films of these compounds have been characterized by their surface pressure versus molecular area (*π*/*A*) and Brewster angle microscopy (BAM) observations.

## 1. Introduction

Various synthetic routes and structural modifications of dendrimers have been proposed, and several of their properties are now understood [1–4]. Therefore, potential applications of dendrimers at surfaces are clearly growing, for example, in surface-based catalysts [5, 6]; in the synthesis and stabilization of metal nanoparticles or as drug delivery supports [7]. Nevertheless, the general principles determining molecular conformation at the air-water interface are still under study [8–10], with unsolved questions such as the differences in the molecular shape near the interface and their relationship to the three-dimensional; the importance of the chemical functionalities of the different parts of the molecule; the shape of the formed monolayers, and so forth. So far, the knowledge of dendrimer structure in Langmuir films (at the air-water interface) comes largely from pressure-area (Π-*A*) measurements, which are however open to multiple interpretations [11–16].

During the past few years there has been a considerable interest in the use of dendrimers as surface and interface active materials. The preparation of Langmuir films from amphiphilic dendrimers or monodendrons has been investigated by several research groups [17–21], since the Langmuir technique helps to access valuable information about the behavior of dendrimers at the air-water interface. Previous publications showed possible approaches to achieve surface activity for dendritic molecules, but few of them have described Langmuir films obtained from dendritic molecules [22–25] and their application.

In this paper, we report the incorporation in Langmuir films of three generations of polyester dendrimers, in order to contribute to the understanding of the supported dendrimer properties and their behavior.

## 2. Experimental

3,5-Dihydroxybenzoic acid (DHBA) (97+%), 1,3,5-tris(2-hydroxyethyl) cyanuric acid (THECA) (97+%), and *p*-toluenesulfonic acid (PTSA) (98.5+%) were purchased from Sigma-Aldrich and used without further purification. Tetrahydrofuran (99+%) was purchased from Sigma-Aldrich, degassed, and dried using standard procedures.

Thin-layer chromatography (TLC) was performed with TLC plates, silica gel on aluminum (Sigma-Aldrich), and Hexane : THF (mix v/v 3 : 2) as an eluent. Column chromatography was performed with Sigma-Aldrich silica gel 60 Å with a pore diameter range of 63–200 *μ*m. ^1^H (TMS at 0.0 ppm internal reference) and ^13^C NMR (DMSO-d_6_ at 39.5 ppm internal reference) experiments were carried out in a 400 MHz NMR spectrometer Varian Unity Inova. Elemental analyses were made on a Fisons EA1108 elemental analyzer. UV-visible absorption spectra, in colloidal dispersion, were taken with an Ocean Optics USB200 miniature fibreglass optic spectrometer and fluorescence spectra were taken with a Jobin Yvon Horiba Fluoromax spectrometer.

**Figure 1:**
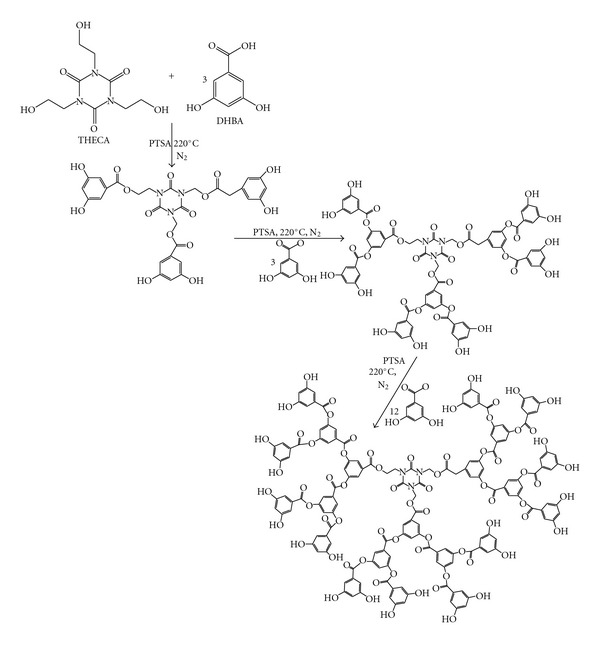
Synthesis of the three G1, G2, and G3 polyester-dendrimer generations.

The hyperbranched polyester dendrimers were produced by the polycondensation of stoichiometric amounts of the monomer DHBA corresponding to each generation with 1 mol of THECA as a trifunctional central core. According to the literature methods [26], the coupling reaction preceded without protection of the hydroxyl groups. The monomer to core ratio was varied between 3 and 12 equivalents. *p*-Toluenesulfonic acid (PTSA) (2 wt% based on DHBA monomer) was used as an acid catalyst in all reactions. The polymerization reaction was carried out in bulk at 220°C. After 10 min, the temperature was reduced to 180°C, the reaction mixture was kept at this temperature for 5 min to obtain several generations of growth, and the procedure was repeated, with a stepwise addition of stoichiometric amounts of monomer to a core molecule in a synthesis designed to mimic the generation of a dendrimer (Figure 1). The yield of all obtained polymers was found to be above 90% for generation 1, and 50% for generations 2 and 3.

G1 was considered to be the first generation after coupling 3 mol of the monomer with 1 mol of the central core. THECA (0.01 mol, 2.61 g), DHBA (0.03 mol, 4.624 g), and PTSA (0.092 g) were placed in a three-necked condensation flask equipped with a nitrogen inlet, a drying tube, and a stirrer. The reaction vessel was evacuated for 10 min and flushed with nitrogen three times and then placed in an oil bath that was preheated to 220°C. The reaction mixture was left to react for 10 min under a stream of alternated nitrogen and vacuum to remove the water formed during the reaction. After the solvent was evaporated, the crude product was purified with column chromatography, resulting in a purified compound in the form of yellow crystals, yield (90%). ^1^H and ^13^C NMR spectroscopic results are consistent with the formulation proposed. Elemental analysis results also agree with the proposed formulation.

For the Langmuir Films, data were collected with a KSV 5000 system 3 using a Teflon trough and barriers in a dust-free environment. The temperature was controlled to ±0.1°C. All the isotherms presented here were taken at 20°C. Ultrapure water (*ρ* = 18.2 MΩ·cm) obtained from a Milli-DIPAK/Milli-Q185 ultrapurification system from Millipore was used for the subphase. Surface pressure was measured by means of a platinum Wilhelmy plate. The spreading solutions were prepared by dissolving the dendrimers in chloroform at 1 mg/mL concentrations. The solutions were spread on the water surface with a microsyringe, and the film was then left for 15–20 min to equilibrate before the compression started. The monolayers were compressed at 4 mm/min. The phase transitions of the spread monolayer were observed using a Nanofilm Technologie Brewster angle microscopy (BAM) fitted with a Teflon trough such that images of the air-solution interface could be taken by the CCD camera. The images were corrected by subtracting a reference image to account for the nonuniform illumination by the laser beam across the image. The image contrast consisted of 256 gray levels.

## 3. Results and Discussion

### 3.1. Synthesis

The dendrimers were synthesized according to common acid-catalyzed esterification procedures [26]. All prepared dendrimers are phenolic-terminated polyesters and they are soluble in methanol, acetone, tetrahydrofuran and DMSO, and partial soluble in chloroform.

The ^1^H NMR (DMSO-d_6_) spectrum shows two sets of signals at 4.4 ppm (2H) and at 3.8 ppm (2H) assigned to the protons on the CH_2_–O groups with another two sets of signals at 4.1 and 3.4 corresponding to the protons on the CH_2_–N groups. Finally three sets of signals at 7.9, 7.8 and 7.4 ppm (9H), were assigned to phenyl protons of benzilic rings. ^13^C NMR (DMSO-d_6_) exhibits a set of various singlets at 160 ppm (2C), at 168 ppm (2C), and 149 ppm (3C) assigned to the carbonyl carbon atoms and to the carbon atoms on the N–C=O, CH_2_ groups, respectively; a second set of signals at 158 ppm (6C), 132 ppm (6C), and 107 ppm (6C) assigned to the aromatic carbon atoms; a third set of signals at 40.9 ppm (3C) and 40 ppm (3C) assigned to the aliphatic carbons. The FAB mass spectrum shows a peak at 669 m/z correspondent to the fragment [*M* ^+^-Cl]. Anal. Calc. for CHN: C, 55.4; H, 4.65; N, 7.18%. Found C, 53.8; H, 4.56; N, 6.96%.

**Figure 2:**
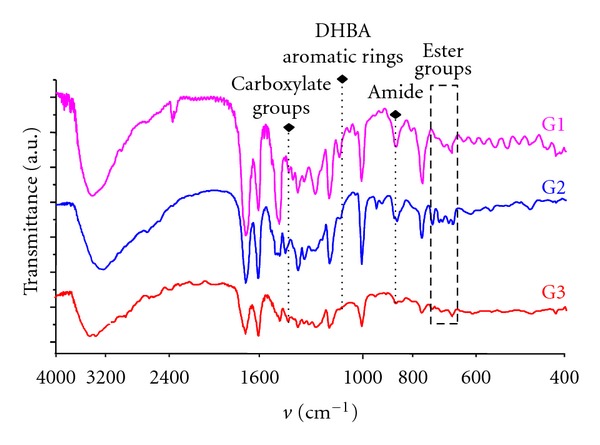
Infrared spectra of G1, G2, and G3 polyester dendrimers.

The IRs of the different generations show slight differences; in the region around 3200 cm^−1^, these differences are attributed to interactions between terminal OH groups, which increases with the generation; thus the band becomes wider. Another area that can be identified as being changed is around 1595 cm^−1^, which corresponds to carboxylate groups; 1200 cm^−1^ which is assigned to DHBA aromatic rings; 900 cm^−1^ corresponding to amide symmetry and anti-symmetry vibrations; 700–650 cm^−1^ to ester groups, which actually are the more affected groups when the generation is growing up. All of these regions are marked in Figure 2.

**Figure 3:**
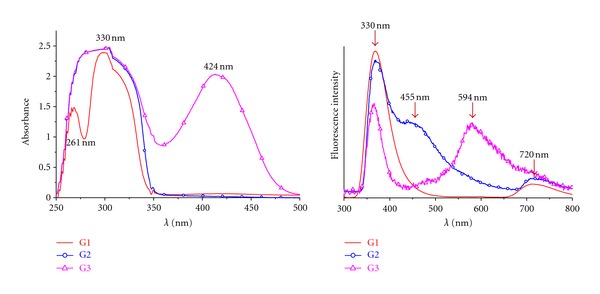
UV-vis absorption and fluorescence emission spectra of three G1, G2, and G3 polyester dendrimer in DMSO at 10^-4 ^M concentration.

The photophysical properties of the obtained dendrimers were studied by UV-vis absorption and fluorescent spectrometry. The UV-vis absorption spectra of the three dendrimers (G1, G2, and G3) in DMSO are shown in Figure 3. The spectrum displays differences between the three dendrimers, with two intense bands at 261 and 330 for G1; a broad band at 330 nm (which includes both the bands of G1) for G2; two intense bands at 330 (again this one include both bands from G1) and at 424 nm for G3. Upon excitation at 260, 330, and 420 nm, correlated with the absorption maxima, the polymers were found to be mainly blue emitters which solutions exhibited intense fluorescence, with two maxima at 330 and 720 nm for G1; at 330, 455 (shoulder) and 720 nm for G2; at 330 and 594 nm, for G3 (Figure 3). The 720 nm emission is less intense and is associated to far-red emitters. As in previous publications [26], both the peak position and the emission profiles remained independent of the excitation wavelength. According to the literature [27], the intense peaks could be attributed to the emission from the intramolecular interaction between excimers. As already suggested, these polymers could be considered fluorescent materials promising for applications in molecular photonics or in fluorescent markers.

### 3.2. Isotherms Surface/Atmospheric Pressure

**Figure 4:**
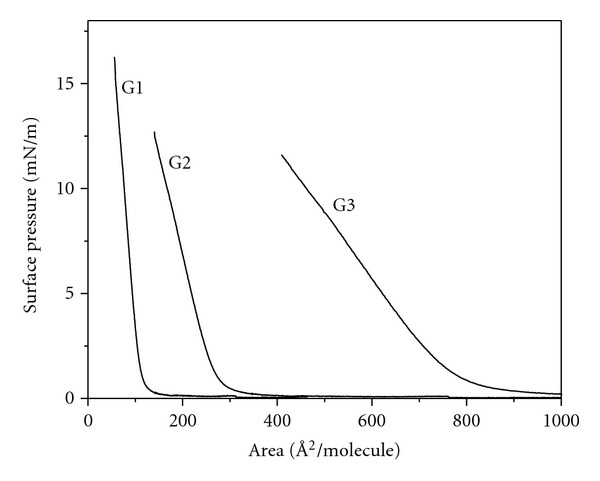
Pressure-area isotherms for G1, G2, and G3 polyester dendrimers.

The LB technique allows measuring changes in surface pressure according to area at constant temperature, in this case 25°C, and with a defined value of absorbed molecules. The isotherms (Figure 4) showed that the polyester dendrimers are dispersed spontaneously over the water-air interphase and that, when compression begins, the film does not collapse, forming Langmuir monolayers even when generation increases; that is, the isotherms showed that it is possible for Langmuir monolayers to be formed with the three polyester dendrimers even though they have a large proportion of hydrophobic aromatic groups, which explains the fact that high surface pressures are not reached.

**Figure 5:**
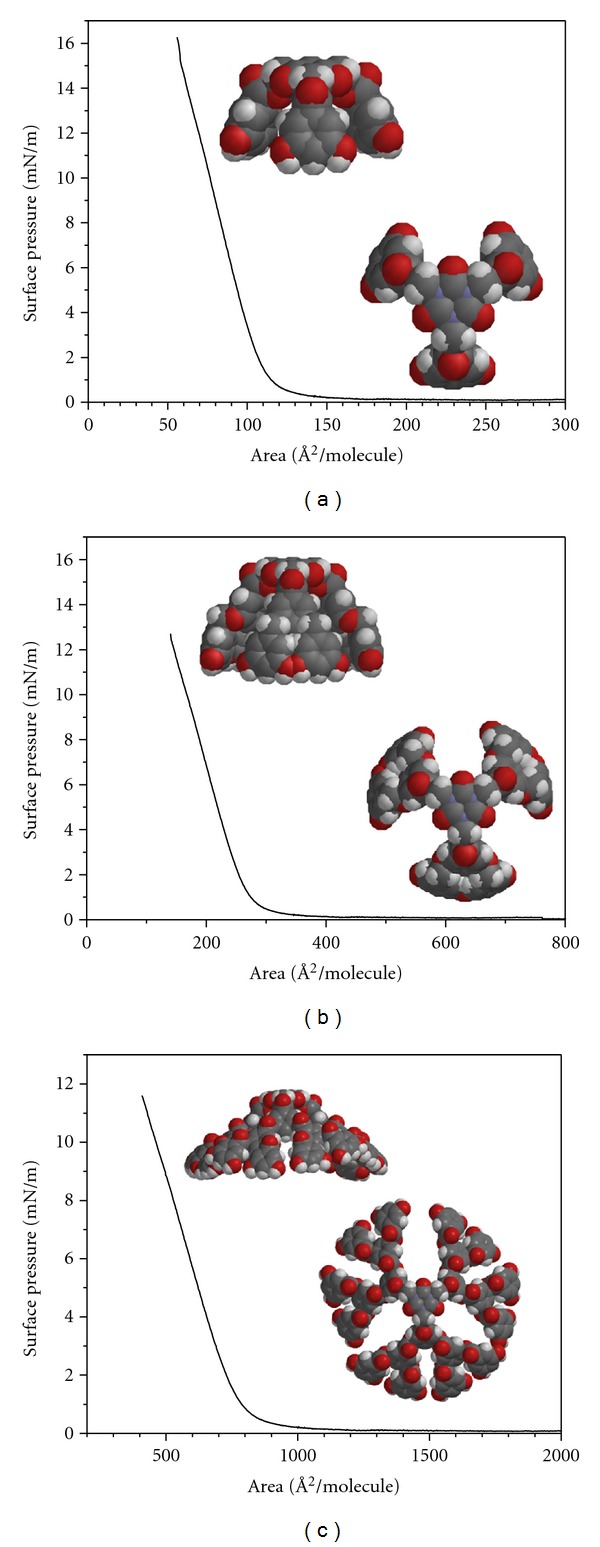
Pressure-area isotherm for polyester-dendrimer generations G1, G2, and G3. Insets: (a) and (b) of the calculated structures of G1, G2, and G3 (molecular modeling performed with Spartan) used to estimate the molecular area.

The collapse pressures that were reached (*π*_*c*_) are summarized in Table 1. The G1 polyester dendrimer reached the highest collapse pressure. The area held by each molecule in the Langmuir monolayer is determined by drawing a tangent over the liquid region of the surface pressure isotherms. The previous graph shows that the G1 polyester dendrimer is the one taking up the smallest area, with 133 Å^2^/molecule, followed by the G2 polyester dendrimer with a molecular area of 291 Å^2^/molecule, whereas the G3 polyester dendrimer held a larger area of 806 Å^2^/molecule. The previous indicates that, when dendrimer generation increases, the molecular area doubles, because molecule size also increases, therefore taking up a greater molecular area. The found areas are close to the expected area for a chain containing phenyl rings [11] and agree perfectly with the molecular modeling calculations (Table 1). G1 has a final molecular area of *A*_0_ = 133 Å^2^, G2, *A*_0_ = 291 Å^2^, and G3, *A*_0_ = 806 Å^2^; for this series, the final molecular area varies almost linearly with the number of OH groups, as can be seen in Figure 5. The final packing must therefore be very dense for G1 molecules and decreases as the dendrimer generation increases.

**Figure 6:**
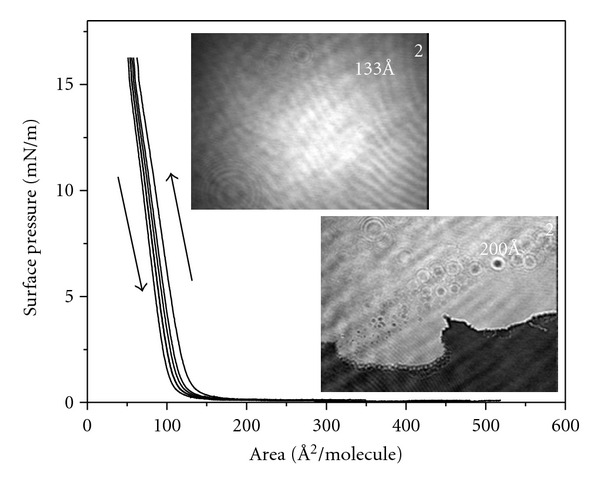
Successive compression/expansion cycles with a monolayer of polyester dendrimer G1 showing the reversibility of the process and Brewster angle microscopy images at *A* = 200 Å^2^ (*π* = 0 ± 0.1 mN/m) and 133 Å^2^ (*π* = 10 ± 0.1 mN/m).

The films for the polyester dendrimer G1 show excellent reversibility in successive compression-expansion cycles, as long as the *π* is kept below the collapse pressure *π*_*c*_ ≈ 16 mN/m, and BAM observations revealed the quality of the films (Figure 6). The film obtained for G1 is noncontinuous at large area values, with holes through it, where water can be seen. These domains smoothly weld together, when the molecular area goes below *A ≈* 133 Å^2^.

For the polyester dendrimers G2 and G3 the behavior is different, that is, showed no reversibility in successive compression-expansion cycles as long as the *π* is kept below the collapse. At the same time, defects can be seen in the BAM pictures confirming that the films are of poor quality, perhaps due to steric hindrance and the imbalance between the hydrophilic and hydrophobic groups with increasing generation of dendrimers [28].

Hysteresis curves of the G1–G3 polyester dendrimers were drawn, making it possible to observe the methods' reproducibility and stability. These were obtained with the same experimental conditions as the isotherms (concentration, barrier speed, injected volume, and temperature), compressing down to a surface pressure lower than that of the collapse, so as to immediately relax the barrier completely and repeat the cycle. 

**Figure 7:**
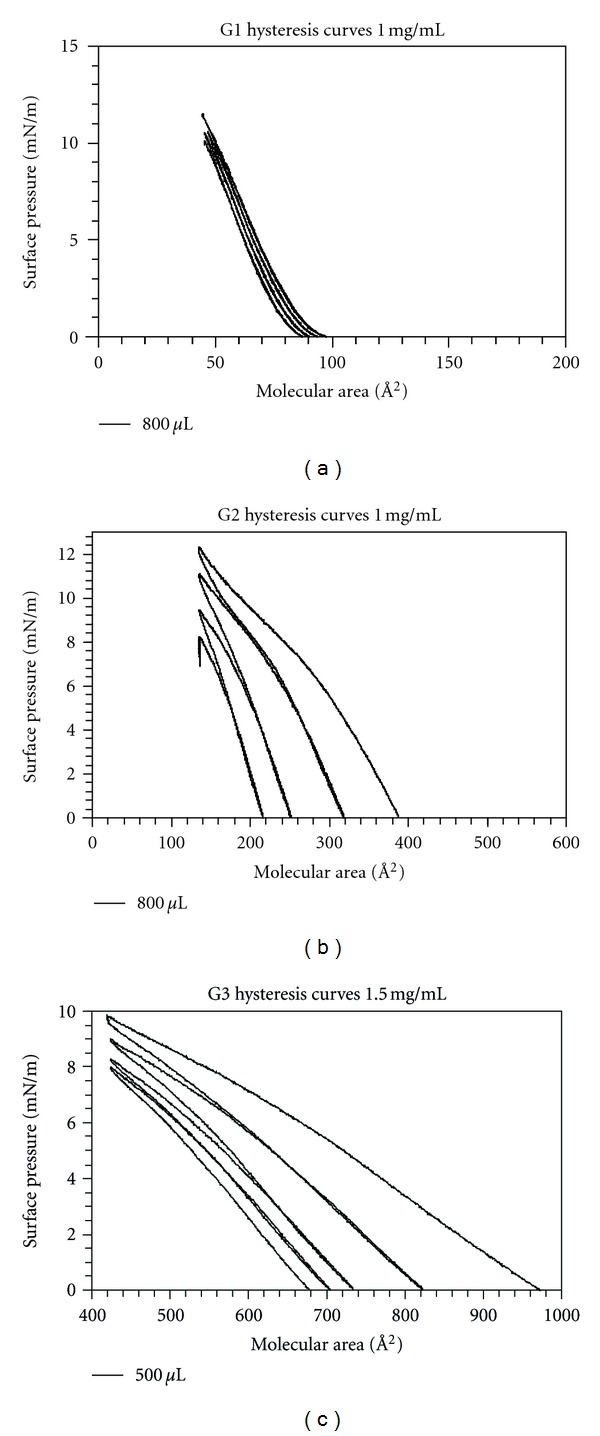
Hysteresis curves for G1, G2, and G3 polyester dendrimers.

The first graph depicted in Figure 7 shows the hysteresis curve of dendrimer G1. It is possible to observe how the compound continues to have the same behavior after 6 cycles of compression and decompression. A very slight and continuous change is observed in the consecutive isotherms toward low molecular area, which is due to the organization that was reached during previous cycles, which is not completely lost. The previous implies that, for G1 polyester dendrimer, as long as compression is made under the same conditions, the same molecule organization will be reached over the water/air interphase of the Langmuir trough.

Comparison of the hysteresis curves (Figure 7) shows that only the G1 polyester dendrimer showed irreversibility, that is, it formed more stable films than dendrimers of higher generation, which confirms that this family of dendrimers is not apt for the transfer of solid supports since it is not possible to maintain equilibrium conditions. Apparently, larger surrounding polyester chains decrease the attraction to the SIO_2_ molecules from the support. The explanation used by Pao et al. [11], for similar results, can be employed here; thus, the hysteresis curves results indicate that the molecular area on larger generations of the studied dendrimers must be largely, but not entirely, determined by the sum of loosely packed individual alkyl chains, with the chains extending radially away from the surface. G1 polyester dendrimer has a somewhat smaller area, but not unphysically so. The limiting molecular area for G1 and G2 is much larger, but the area after compression to the plateau is again in the same range. A provisional explanation for this effect could be that the molecule lies essentially in a “flat” way on the surface in the gas phase, but lifts off and extends under compression.

### 3.3. Brewster Angle Microscopy (BAM)

**Figure 8:**
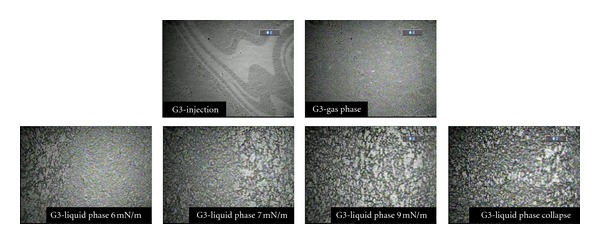
Interfacial behavior of the G3 polyester dendrimer at 25°C, 1.5 mg/mL, 600 *μ*L.

Figure 8 shows a series of images that are representative of the interfacial behavior of the G3 polyester dendrimer, in a wide range of areas between 806 and 133 Å^2^/molecule. The first image shows the interface during the injection process, when the solvent is still present. It is possible to observe two shades, the clearer one being the solvent, which in this case is water. The second image shows the film aspect during the compression process, where it is possible to observe that the film is homogeneous. The third image, which corresponds to a surface pressure of 6 mN/m, shows the emergence of a liquid phase. In the following images, it is possible to observe the emergence of domains that extend over the surface as pressure increases, until reaching the collapse pressure, at which monolayer rupture occurs and structural systematic defects appear.

**Figure 9:**
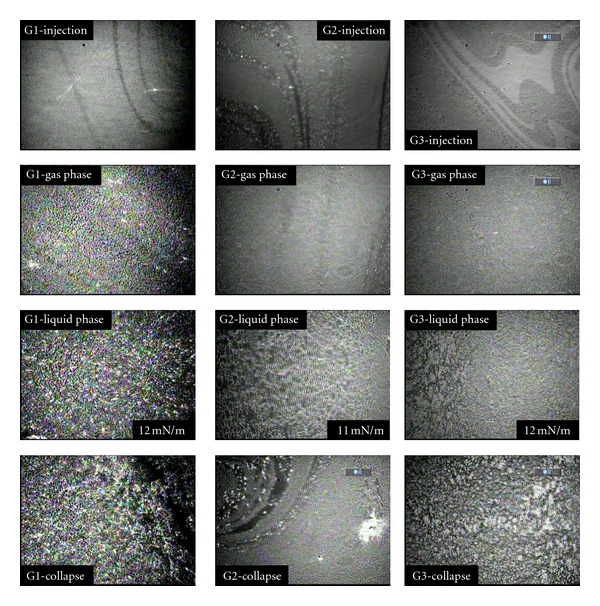
Effect of generation on interfacial structure for THECA dendrimers 1 mg/mL, 1000 *μ*L.

In a comparison of BAM images for the polyester dendrimers (Figure 9), the effect of generation on molecular orientation in the water/air interface can be seen. Considering the variations in contrast between experiments, a similar molecular arrangement is observed in the gas phase for all three dendrimers. Subsequently, it is possible to detect how the domains coalesce into a homogeneous liquid phase in the following series of images. Finally, it is shown that the structural defects are more important, proportional to the generation. 

## 4. Conclusions

In conclusion, we have shown that the polyester dendrimers are suitable amphiphilic derivatives for the preparation of Langmuir films; however, the quality of the films decreases with increasing dendrimer generation, which suggests that larger dendrimers are not apt for the transfer of solid supports since it is not possible to maintain equilibrium conditions. The amphiphilic character of G2 and G3 polyester dendrimers might be reduced due to the relatively low hydrophilicity of the central triazine-2,4,6-trione group. This may result in films that are relatively unstable to multilayer collapse compared with the small generation dendrimer studied. Thus we are currently working on the synthesis of composites with the polyester dendrimers and Pd(0) NPs, to study their modification on the solid support transference. As expected, it can be observed that the surrounding polyester chains' length of the dendritic branches has a remarkable influence on the isotherm phases as well as on the packing of highest generation dendrimers in the monolayers. The studies on the composites, polyester-dendrimers-Pd(0) NPs will conduce on the transference of the Langmuir monolayers onto solid substrates, using the LB technique to study their catalytic reactions on heterogeneous catalysis.

## Figures and Tables

**Table 1:** Langmuir isotherms parameters for the molecules (recorded at 20°C).

Polyester dendrimers	Concentration (mg/mL)	Injected volume (mL)	Molecular weight (g/mol)	*A*_*o*_ (Å^2^)_Experimental_	*A*_*o*_ (Å^2^)_Molecular modeling_	*π*_*c*_ (mN/m)
G1	1.0	1.0	655.52	133	139	16
G2	1.0	1.0	1471.15	291	293	12
G3	1.0	1.0	3105.41	806	810	11
